# Häufig gestellte Fragen (FAQ) in der Risikokommunikation zu COVID-19: Erstellung und Bedeutung als interinstitutionelles Krisenreaktionsinstrument

**DOI:** 10.1007/s00103-022-03532-z

**Published:** 2022-04-13

**Authors:** Linda Seefeld, Florentine Frentz, Nina Horstkötter, Christoph Peter, Martin Dietrich

**Affiliations:** Referat „Infektionsschutz, Risiko- und Krisenmanagement“, Bundeszentrale für gesundheitliche Aufklärung, Köln, Deutschland

**Keywords:** Gesundheitsaufklärung, BZgA, Krisenkommunikation, Krisenmanagement, Coronavirus, SARS-CoV‑2, Pandemie, Health education, Federal Center, Risk communication, Crisis management, Coronavirus, SARS-CoV‑2, Pandemic

## Abstract

In der Ergänzung des Nationalen Pandemieplans zur Bewältigung der COVID-19-Pandemie ist festgelegt, dass die Bundeszentrale für gesundheitliche Aufklärung (BZgA) über die Internetseite www.infektionsschutz.de Informationsmaterial zum Coronavirus SARS-CoV‑2 für die Allgemeinbevölkerung zur Verfügung stellt. Dieses soll insbesondere Antworten auf häufig gestellte Fragen (FAQ) sowie Verhaltensempfehlungen zur Prävention beinhalten.

Dieser Artikel beschreibt, wie die Ad-hoc-Erstellung von Informationsinhalten in Form von FAQ erfolgt und welche Bedeutung diese in der Krisenkommunikation haben. Dabei wird der Wandel der FAQ vom einfachen Informationsangebot zum interinstitutionellen Krisenreaktionsinstrument (Rapid Reaction Tool) im Rahmen einer agilen Kommunikation zum Coronavirus deutlich. Im Sinne einer kongruenten und tagesaktuellen Informationsbereitstellung ist eine enge Zusammenarbeit zwischen den Institutionen erforderlich. Die Arbeits- und Abstimmungsprozesse sowie verschiedene Vorgehensweisen bei der Aktualisierung werden vorgestellt.

Aus den beschriebenen und bewerteten Arbeitsprozessen können theoretische Implikationen für die Krisenkommunikation und das Krisenmanagement – insbesondere die Zusammenarbeit zwischen verschiedenen Institutionen – abgeleitet werden. Auch können sie von anderen Institutionen als Beispiel für „gute Praxis“ aufgegriffen und ggf. weiterentwickelt und auf andere Kontexte übertragen werden.

## Abstract (en)

In addition to the national pandemic plan to cope with the COVID-19 pandemic, it is stipulated that the Federal Centre for Health Education (BZgA) provides information on coronavirus SARS-CoV‑2 for the general population via a subpage of www.infektionsschutz.de. In particular, the informational material should contain answers to frequently asked questions (FAQ) as well as behavioural recommendations for prevention.

This article describes how information content is created ad hoc in the form of FAQ and why these FAQ are significant for crisis communication. The evolution of the FAQ from a simple information instrument to an inter-institutional rapid reaction tool in the context of risk communication on COVID-19 becomes clear. Close cooperation between the authorities is required to ensure that information is provided in a congruent and up-to-date manner. The work and coordination processes as well as various update procedures are presented. Theoretical implications for crisis communication and crisis management can be derived from the work processes described and assessed.

These processes can be taken up by other institutions as examples of good practice and, if necessary, further developed and/or transferred to other contexts.

## Die Rolle der Bundeszentrale für gesundheitliche Aufklärung (BZgA) bei der Krisenkommunikation in der Coronapandemie

Weltweit hat das Coronavirus SARS-CoV‑2 eine ernste gesundheitliche Krisensituation hervorgerufen. Die rasante Ausbreitung von COVID-19 wurde am 30.03.2020 von der Weltgesundheitsorganisation (WHO) zur Pandemie erklärt [1]. Aufgrund der Ausbreitung hierzulande hat der Deutsche Bundestag mit Wirkung zum 28.03.2020 eine epidemische Lage von nationaler Tragweite und unbestimmter Dauer festgestellt [2]. Die Lage wurde wiederholt bestätigt [3], ist mit Ablauf des 25.11.2021 ausgelaufen und wurde durch ein Gesetz zur Änderung des Infektionsschutzgesetzes und weiterer Gesetze abgelöst [4].

Mit Stand vom 20.12.2021 wurden in Deutschland insgesamt ca. 6,8 Mio. COVID-19-Fälle gemeldet sowie mehr als 108.000 Todesfälle an und mit COVID-19 [5]. Weltweit registrierte die WHO bis zu diesem Zeitpunkt rund 272 Mio. COVID-19-Fälle und mehr als 5,3 Mio. darauf zurückgeführte Todesfälle [6]. Damit stellt die COVID-19-Pandemie die bisher größte gesundheitspolitische Herausforderung dieses Jahrhunderts dar [7, 8]. Die erforderliche Krisen- und Risikokommunikation ist essenzieller Bestandteil dieser Herausforderung, denn Information und Kommunikation werden mitunter als die wirksamsten präventiven Maßnahmen in der Pandemie bezeichnet [9]. Ziele der Risikokommunikation sind, (1) die Bevölkerung zu sensibilisieren, (2) Informationen zu einem adäquaten Umgang mit Risiken zu vermitteln, (3) Dialog und Konsens über das Risiko zwischen verschiedenen Anspruchsgruppen herzustellen, (4) soziale Beziehungen zu relevanten Ziel- bzw. Dialoggruppen aufzubauen, (5) Werte zu vermitteln und zu verankern, (6) Akzeptanz für Entscheidungen zu generieren sowie (7) Verhaltensänderungen auszulösen [10]. Übergeordnet stehen einerseits Aufbau, Pflege und Stärkung von Vertrauen in die Informationen sowie die Entscheidungsträger und -vermittler und andererseits der Abbau von Ängsten und Skepsis [8, 11–13].

Die Allgemeinheit über die Risiken übertragbarer Krankheiten und die Möglichkeiten zu deren Verhütung zu informieren, ist eine öffentliche Aufgabe (Infektionsschutzgesetz, IfSG § 3; [14]), mit deren Erfüllung auf nationaler Ebene insbesondere die Bundeszentrale für gesundheitliche Aufklärung (BZgA) betraut ist. Die BZgA ist eine Obere Bundesbehörde im Geschäftsbereich des Bundesministeriums für Gesundheit (BMG). Ihre Aufgabe ist es, in enger Abstimmung mit dem BMG und je nach Fragestellung mit den Schwesterbehörden ein informiertes, verantwortungsbewusstes und gesundheitsgerechtes Verhalten der Bevölkerung zu fördern und diese zu motivieren und darin zu unterstützen, die Angebote des Gesundheitssystems sachgerecht zu nutzen. Die BZgA leistet u. a. einen Beitrag zur Entwicklung und Umsetzung nationaler Aktionspläne und Programme sowie bei gesetzlichen Aufgaben [15].

Durch die Überarbeitung des Nationalen Pandemieplans (2017) wurde die Rolle der BZgA im Bereich der bevölkerungsweiten Risiko- und Krisenkommunikation gestärkt. Es ist festgelegt, dass die BZgA in Zusammenarbeit mit anderen Bundesbehörden im Falle einer Pandemie mit der „Realisierung bundesweit ausgerichteter Kommunikationsmaßnahmen für die Bevölkerung“ betraut ist [16, 17]. Zudem wurde bereits 2013 die Rolle der BZgA wie auch des Robert Koch-Instituts (RKI) und des BMG in der Risiko- und Krisenkommunikation in Form einer Verwaltungsvorschrift festgelegt [17]. Konkret wird für die BZgA die Bereitstellung von Informationsmaterial im Internet für die Bevölkerung sowie Multiplikatorinnen und Multiplikatoren im Bereich Prävention und Gesundheitsförderung auf Grundlage der Fachinformationen des RKI genannt. Das Informationsmaterial soll insbesondere Antworten auf häufig gestellte Fragen (sogenannte Frequently Asked Questions – FAQ) sowie Verhaltensempfehlungen zur Prävention beinhalten. Bei diesen FAQ gilt es stets, die Bedarfe der Bevölkerung zu berücksichtigen und gleichzeitig Aktualität zu gewährleisten. In der „Ergänzung zum Nationalen Pandemieplan – COVID-19 – neuartige Coronaviruserkrankung“ ist festgelegt, dass diese Informationen über eine Unterseite von infektionsschutz.de veröffentlicht werden sollen [18].

FAQ stellen für die BZgA die am schnellsten umsetzbare Möglichkeit dar, ein Informationsangebot aufzubauen, welches die Öffentlichkeit, Multiplikatorinnen und Multiplikatoren sowie spezifische Zielgruppen (z. B. ältere Menschen, Eltern Minderjähriger, Reisende) im Rahmen der Krise in Echtzeit erreichen kann [19–21]. Da die Verwendung digitaler Endgeräte verbreitet ist, ist davon auszugehen, dass die FAQ für die meisten Menschen niedrigschwellig nutzbar sind [22].

Die FAQ sind aber nicht nur Informationsquelle für die Rezipienten, sondern auch inhaltlicher Ausgangspunkt für die Entwicklung weiterer BZgA-Angebote. Die BZgA hat ein umfangreiches Repertoire an Materialien und Medien zum Coronavirus SARS-CoV‑2 mit Verhaltensempfehlungen zur aktuellen Lage zusammengestellt, um Menschen in ihren Lebenswelten bestmöglich zu erreichen (u. a. Themenseiten, Merkblätter, Checklisten, Infografiken, Broschüren, Beiträge in den audiovisuellen (AV) und sozialen Medien; [23]).

Von Rüden et al. [20] stellen die bedarfsbezogene Kommunikationsstrategie der BZgA während der COVID-19-Pandemie dar und erläutern, wie Zielgruppen informiert sowie Multiplikatorinnen und Multiplikatoren eingebunden werden. Die Erstellung und Bedeutung von FAQ in der Krisenkommunikation wird dabei jedoch nicht im Detail beschrieben. Die Darstellung des konkreten Vorgehens bei der Entwicklung und Veröffentlichung der FAQ kann zu mehr Transparenz und einem besseren Verständnis der Rolle der BZgA in der Krisenkommunikation und zur Stärkung des Vertrauens in die Behörde beitragen.

Dieser Artikel beschreibt die Ad-hoc-Erstellung von Informationsinhalten in Form von FAQ und die Weiterentwicklung dieses Prozesses im Laufe der Pandemie. Dabei wird der Wandel der FAQ vom einfachen Informationsangebot zum interinstitutionellen Krisenreaktionsinstrument (Rapid Reaction Tool) und dessen Bedeutung im Rahmen einer agilen Kommunikation zur Coronapandemie verdeutlicht. Die intra- und interinstitutionellen Arbeits- und Abstimmungsprozesse sowie verschiedene Vorgehensweisen bei der Aktualisierung werden vorgestellt.

Aus den beschriebenen Arbeitsprozessen können theoretische Implikationen für die Krisenkommunikation und das Krisenmanagement – insbesondere die Zusammenarbeit zwischen verschiedenen Institutionen – abgeleitet werden. Auch können sie von anderen Institutionen als Beispiel für „gute Praxis“ aufgegriffen und ggf. weiterentwickelt sowie auf andere Kontexte übertragen werden.

Im Folgenden werden die zugrunde liegenden Prozesse von Beginn der Pandemie bis heute erläutert, verglichen und bewertet.

## Chronologische Entwicklung der von der BZgA bereitgestellten FAQ zu COVID‑19

**Figure 1:**
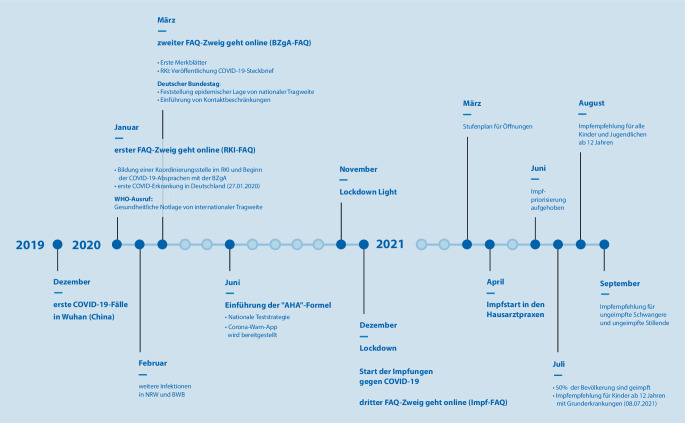

Ende 2019 treten in Wuhan (China) erste Fälle einer unbekannten Lungenerkrankung auf; dies markiert den Beginn der Pandemie (Abb. 1). Die BZgA wird über die Epidemiologische Lagekonferenz (EpiLag^1^) informiert und erstellt intern erste Grundlageninformationen. Am 22.01.2020 nimmt das RKI Kontakt zum Leiter des Krisenstabs der BZgA sowie dem für den Infektionsschutz zuständigen Fachreferat^2^ auf und informiert die interne Koordinierungsstelle. Das weitere Vorgehen zur Kommunikation wird abgestimmt. Einen Tag später veröffentlicht die BZgA auf ihrer Startseite (bzga.de [24]) eine Meldung zu Informationen zum neuartigen Coronavirus auf infektionsschutz.de [25] und setzt 2 Tweets zum Thema „Schutz vor Virusinfektionen“ (ohne direkten Bezug zum Coronavirus) ab. Im Januar 2020 veröffentlicht das RKI erste FAQ. Die BZgA extrahiert die für die Bevölkerung relevanten Fragen, bereitet diese allgemeinverständlich auf und stellt sie zur Verfügung. Für den weiteren Verlauf wird mit der RKI-Pressestelle ein Prozedere zur zeitnahen Aktualisierung vereinbart.

Ende Januar 2020 klassifiziert die WHO das Auftreten des Coronavirus als „gesundheitliche Notlage von internationaler Tragweite“ [1]; einen Tag später schaltet Twitter einen automatischen Verweis auf die Informationen der BZgA zum Coronavirus.

Im Februar 2020 treten weitere Infektionen in Deutschland auf [26, 27]. Die FAQ der BZgA werden ausgebaut und u. a. als Merkblatt (PDF) zur Verfügung gestellt sowie ergänzend als Erklärvideos unter dem Titel „Corona Wissen kompakt“ für das Internetvideoportal Youtube aufbereitet [28]. In den Wochenendausgaben überregionaler Tageszeitungen werden Anzeigen mit Informationen zum Coronavirus mit Verweis auf infektionsschutz.de [25] geschaltet. Die für die BZgA tätige Kommunikationsagentur^3^ wird in den Krisenmodus versetzt (verkürzte Reaktionszeiten inklusive Einsatzbereitschaft in den Abendstunden sowie am Wochenende), um eine schnelle und mit einem externen Redaktionsteam koordinierte Anpassung der Inhalte auf der Website zu gewährleisten.

Ab März 2020 stellt das RKI einen epidemiologischen Steckbrief zu SARS-CoV‑2 und COVID-19 [29] zur Verfügung und verweist in einigen FAQ darauf. In diesem Zuge entwickelt die BZgA eigene FAQ als Ergänzung zu den vom RKI erstellten FAQ (RKI-FAQ), welche auf den Informationen des Steckbriefs des RKI [29] und auf Anfragen der Bevölkerung beruhen sowie auf aktuellen Anforderungen, die sich z. B. aus Presseberichten und -anfragen oder auf Bitten des BMG ergeben. Auch hierzu wird ein Vorgehen der gegenseitigen Bereitstellung von Informationen und gemeinsamen Abstimmung mit dem RKI vereinbart. Zudem wird eine Kooperation mit dem Institut für Qualität und Wirtschaftlichkeit im Gesundheitswesen (IQWiG) etabliert, das bei einigen fachlichen Fragen unterstützt und ebenfalls FAQ zur Verfügung stellt. Durch den Zuwachs an Informationen bzw. die steigende Menge an FAQ wird für andere Institutionen eine technische Möglichkeit zur Einbindung der FAQ in ihre Websites (iFrame [30]) geschaffen. Zudem werden die FAQ zur besseren Übersichtlichkeit und Auffindbarkeit auf der Website in themenspezifische Rubriken (z. B. „Ansteckung und Übertragung“, „Ausbreitung des Virus“, „Sich und andere schützen“ etc.) geclustert. Im Sommer 2020 werden erste FAQ zum Thema Impfen veröffentlicht – zunächst nur zum Impfen allgemein. Sie sind zu diesem Zeitpunkt Bestandteil der BZgA-FAQ.

Im Dezember 2020 werden bundesweite Kontaktbeschränkungen ausgesprochen [31]. Ende des Monats erfolgt die Zulassung des ersten Impfstoffs gegen COVID-19 [32]. Zur Gewährleistung einer umfassenden und zielgenauen Kommunikation wird auf Bundesebene ein Steuerungskreis „Servicestelle Corona-Impfdialog“ eingerichtet. Dieser setzt sich aus Mitgliedern des BMG, des RKI (welches das Paul-Ehrlich-Institut (PEI) einbezieht), der BZgA und beteiligten Agenturen zusammen und gibt u. a. Hinweise auf zeitnah neu zu entwickelnde FAQ zur Coronaschutzimpfung. Die Kommunikation zur COVID-19-Impfung wird durch das BMG gesteuert. Der Steuerungskreis stimmt die Kommunikation und somit auch die Kommunikationsschwerpunkte zwischen den genannten Institutionen ab [33].

Das BMG verantwortet mit inhaltlicher Unterstützung vom RKI und der BZgA die Webseite zusammengegencorona.de [34], wo weitere FAQ von RKI und BMG zur Verfügung gestellt werden. Die BZgA etabliert aufgrund der stetig steigenden Anzahl an impfbezogenen FAQ sowie der Zulassung eines Impfstoffs gegen COVID-19 weitere Impf-FAQ. Für die Umsetzung und Aktualisierung der Impf-FAQ wird ein neues Vorgehen zur zunächst sehr engen gegenseitigen Information und Abstimmung vereinbart. Mit dem Stufenplan der Öffnungen von Bund und Ländern (März 2021; [35]) werden die FAQ zur Coronaschutzimpfung erweitert.

## FAQ-bezogene Arbeits- und Abstimmungsprozesse

Die Anforderungen an FAQ-Inhalte und die Bedingungen für deren Effektivität, Effizienz und Benutzerfreundlichkeit sind in Infobox 1 dargestellt. Aspekte, die sich bei der Erstellung und Aktualisierung von FAQ zur Coronakommunikation im Sinne von „guter Praxis“ als wichtig erwiesen haben, finden sich in Tab. 1. Im Folgenden wird näher auf den Erstellungsprozess der FAQ eingegangen.

**Table 1:** 

Bereich	Gute Praxis bei der Erstellung und Aktualisierung von FAQ
*Organisation*	Festlegung von Abläufen und Zeiten
	Festlegung von Aktualisierungszyklen
	Vereinbarungen für Rufbereitschaften und technische sowie fachliche Unterstützung (am Wochenende, an Feiertagen oder in den Abendstunden)
	Festlegung von Zuständigkeiten für spezifische Themen- bzw. FAQ-Bereiche
	Austausch zwischen den Beteiligten aller FAQ-Bereiche sowie regelmäßige Meetings, in denen grundlegende sowie aktuelle Punkte abgesteckt und besprochen werden
*Qualitätssicherung*	Verwendung von Checklisten zum Abhaken und zur Dokumentation
	Erstellung/Nutzung eines Redaktionsleitfadens (z. B. Vermeidung von Fachbegriffen und Nutzung von festgelegten allgemeinverständlichen Synonymen): Dieser ist hilfreich bei wechselnden personellen Zusammensetzungen und trägt zur Einheitlichkeit/Allgemeinverständlichkeit der Formulierungen bei
	Im Falle von heterogenen Informationen verschiedener Institutionen: Vereinbarung von Sprachregelungen untereinander
	Umsetzung aller inhaltlich relevanten Schritte zur Qualitätssicherung im Vieraugenprinzip
	Kenntlichmachen aktualisierter Änderungen (z. B. aufgrund neuer Erkenntnisse) durch Angabe des Datums der letzten Anpassung jeweils unter der Antwort sowie der entsprechenden Unterkategorie
*Zielgruppenansprache*	Bei der Auswahl der FAQ für die Allgemeinbevölkerung: Fokussierung auf die Alltagsrelevanz und Verständlichkeit verschiedener Zielgruppen
*Datenquellen und Methoden*	Nutzung verschiedener Methoden und Datenquellen, um die Perspektiven der Allgemeinbevölkerung zu integrieren (z. B. Ergebnisse aus Bevölkerungsbefragungen, Monitoring von Bürgerinnen- und Bürgeranfragen, Befragungen von Beratungs- und Gesundheitsfachkräften, fachübergreifende Expertinnen- und Expertenhearings, Social-Media-Monitoring)
*Information*	Anbieten von/Verlinken zu weiterführenden Informationen
	Identifizierung/Formulierung möglicher Handlungsempfehlungen, konkreter Vorgehensweisen sowie Benennung geeigneter Ansprechpartner bzw. Institutionen
	Transparente Nennung fehlender Informationen bzw. mangelnder wissenschaftlicher Evidenz oder unzureichender Datengrundlage, um Vertrauen zu schaffen

**Figure 2:**
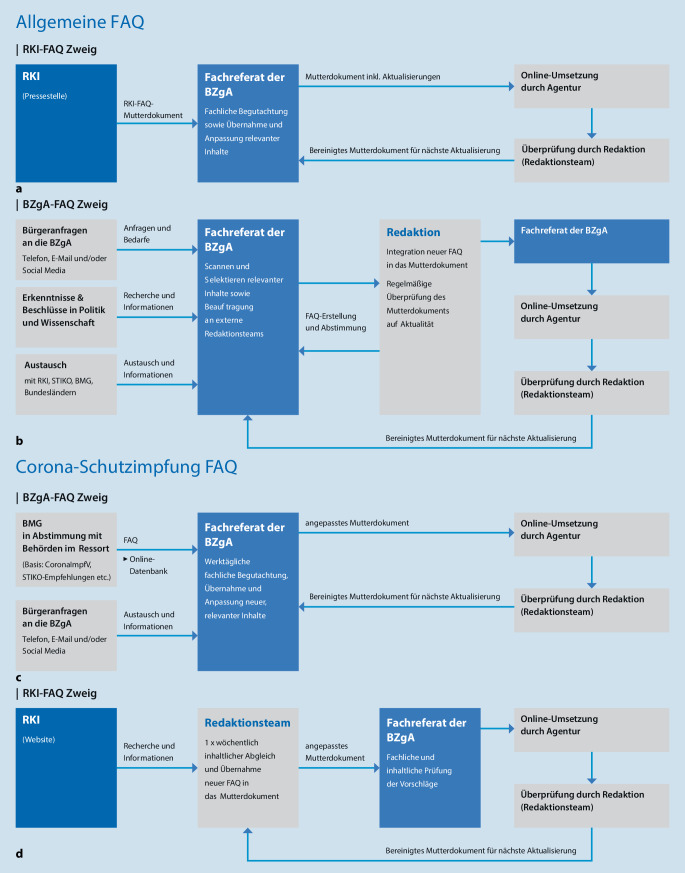

Durch die dynamische Lage und damit einhergehende Informationsbedarfe haben sich zwei FAQ-Bereiche entwickelt: (1) allgemeine FAQ und Coronaschutzimpfungs-FAQ (Abb. 2). Diese Unterteilung basiert auf den verwendeten Quellen und thematischen Unterschieden. Sie dient der besseren internen Arbeitsteilung sowie einem effektiven Projektmanagement und spiegelt nicht die Kategorisierung der FAQ auf der Website wider. Die thematische Aufteilung mittels Unterkategorien auf der Webseite infektionsschutz.de [25] orientiert sich an der Benutzerfreundlichkeit und den Informationsbedarfen der Nutzerinnen und Nutzer. So gibt es beispielsweise FAQ, die sich an bestimmte Zielgruppen (z. B. Eltern, Schwangere, Stillende oder Reisende) richten oder bestimmte Themenschwerpunkte aufgreifen wie die aktuellen Schutzmaßnahmen [20, 36, 40]. An geeigneten Stellen wird innerhalb der FAQ auf Inhalte des RKI, Internetportale des BMG oder Materialien und Seiten anderer relevanter Einrichtungen (z. B. PEI, Bundesinstitut für Arzneimittel und Medizinprodukte (BfArM), WHO) verlinkt. Dabei wird im Sinne der One Voice Policy [41] darauf geachtet, dass die Botschaften der verschiedenen Absender kongruent sind.

Die beiden FAQ-Bereiche werden jeweils von einem Team betreut. Beide Teams werden von einem externen Redaktionsteam sowie einer externen Kommunikationsagentur unterstützt. Das externe Redaktionsteam ist für die Erstellung und regelmäßige Aktualisierung von leicht verständlichen FAQ sowie die Qualitätssicherung zuständig.^4^ Dazu zählen auch die Beobachtung aktueller Entwicklungen und die Identifikation relevanter Themen. Die für die BZgA tätige Kommunikationsagentur ist für die Online-Umsetzung der FAQ zuständig (im Folgenden Agentur genannt)^5^.

### Allgemeine FAQ

#### RKI-FAQ.

Das RKI stellt einen umfassenden Pool an FAQ zum Thema Coronavirus SARS-CoV‑2 bzw. COVID-19 auf seiner Internetseite zur Verfügung [42], der sich vor allem an die Fachöffentlichkeit richtet. Sobald es Aktualisierungen an diesen Inhalten gibt, werden diese von der RKI-Pressestelle an das entsprechende Fachreferat in der BZgA übermittelt (Abb. 2a). Dieses prüft, ob die Änderungen für die Allgemeinbevölkerung relevant sind und übernimmt Inhalte bei Bedarf. Sofern notwendig, gibt es direkte Abstimmungsschleifen zwischen dem Fachreferat und der RKI-Pressestelle, um FAQ zu optimieren oder neue FAQ anzustoßen. Wichtige Inhalte werden zusätzlich beworben, z. B. von der Pressestelle der BZgA durch unterstützende Tweets.

#### BZgA-FAQ.

Die BZgA-FAQ werden ad hoc durch die BZgA formuliert (Abb. 2b). Die Bedarfe der Bevölkerung werden dabei unterschiedlich ermittelt. Sie speisen sich zum Großteil aus den bereits durch von Rüden et al. beschriebenen Datenquellen der BZgA sowie dem Austausch mit anderen Institutionen [42]. Zudem werde Inhalte und FAQ diverser anderer Bundesbehörden einbezogen. Eine umfassende Übersicht zu den inhaltlichen Grundlagen der BZgA-FAQ findet sich in Infobox 2.

Das Fachreferat entscheidet, welche FAQ aufbereitet werden sollen (Abb. 2b). Nach Erarbeitung eines ersten Formulierungsvorschlags greift ein standardisiertes Verfahren, in dessen Rahmen die FAQ anhand bestimmter Kriterien geprüft, ggf. verbessert und online gestellt werden. Für die regelmäßige Überprüfung auf Aktualität werden die FAQ in einem Mutterdokument vom Redaktionsteam mit den genutzten Quellen abgeglichen. Notwendige Aktualisierungen oder Ergänzungen werden im Mutterdokument kenntlich gemacht und durch das Fachreferat und die Redaktion bearbeitet.

### Coronaschutzimpfungs-FAQ

#### BZgA-FAQ.

Um die Bevölkerung korrekt und einheitlich zur Coronaschutzimpfung zu informieren, werden die betreffenden FAQ des BMG, des RKI, des PEI und der BZgA aufeinander abgestimmt (Abb. 2c). Die Basis für diese Abstimmung bildet in erster Linie eine Onlinedatenbank (SABIO®); eine browserbasierte Wissensdatenbank, in der Inhalte zentral gespeichert und gepflegt werden und aus der Daten schnell abgerufen werden können. Die Datenbank wurde im Dezember 2020 für das Themenfeld Corona und Coronaschutzimpfung im Auftrag des BMG aufgesetzt. Sie beinhaltet sämtliche FAQ von zusammengegencorona.de [31]. Zu den Nutzergruppen zählen sowohl die Institutionen BMG, RKI, BZgA und PEI sowie Dienstleister (Agenturen) und Mitarbeitende von Callcentern (116 117, Gebärden-Videotelefonie auf zusammengegencorona.de). Die Datenbank ermöglicht einen gemeinsamen, transparenten Austausch und die Verfügbarkeit von Inhalten in Echtzeit.

Das FAQ-Team für die Coronaschutzimpfungs-FAQ überprüft in Sabio® in einem täglichen Aktualisierungsprozess, welche FAQ auf zusammengegencorona.de [34] hinzugekommen sind oder verändert wurden und welche den Bedarfen der Allgemeinbevölkerung entsprechen. Zur Orientierung dienen hier Auswertungen der schriftlichen und telefonischen Anfragen an die BZgA. Relevante Inhalte werden in das interne Mutterdokument überführt und angepasst. Falls Bürgeranfragen an die BZgA durch diese FAQ nicht abgedeckt werden, werden weitere FAQ von der BZgA formuliert oder bestehende FAQ erweitert.

#### RKI-FAQ.

Neben dem täglichen Aktualisierungsprozess existiert ein wöchentlicher Abgleich, im Rahmen dessen das Redaktionsteam die FAQ des RKI zur Coronaschutzimpfung [48] auf Aktualisierungen überprüft und mit den FAQ der BZgA abgleicht (Abb. 2d). Für diesen Prozess gibt es ein weiteres Mutterdokument, welches dem Fachreferat übermittelt wird.

### Erfahrungen und daraus resultierende Prozesse – die FAQ-Bereiche im direkten Vergleich

Die beiden vorgestellten FAQ-Bereiche „allgemeine FAQ“ und „Coronaschutzimpfungs-FAQ“ haben insbesondere aufgrund ihres Umfangs und ihrer zugrunde liegenden Kooperationen unterschiedliche Bearbeitungsprozesse.

Für die allgemeinen FAQ (Abb. 2a, b) haben sich die Nutzung von Mutterdokumenten und die Prozesse zur Aktualisierung mittels Standardprogramme zur Textverarbeitung und Tabellenkalkulation bewährt. Bei den FAQ zur Coronaschutzimpfung wurden jedoch nach einer kurzen Testphase die Nachteile eines durch mehrere Behörden genutzten und zu pflegenden Dokuments deutlich (u. a. doppelte (Mutter‑)Dokumente durch zeitgleiche Überarbeitungen und lange Abstimmungswege). Zwecks besserer Koordination und Organisation werden die FAQ aller Beteiligten daher nun zentral in einer Datenbank erfasst: Die Datenbank ermöglicht eine schnelle Aktualisierung und Abstimmung sowie die Kategorisierung einzelner Themenfelder und eine spezifische Suche nach bestimmten Inhalten. Durch verschiedene Zugriffsrechte können Benutzergruppen ihre Rollen wahrnehmen und entsprechend Inhalte bearbeiten. Filtereinstellungen ermöglichen es, dass dem Team nur freigegebene und aktuellste FAQ angezeigt werden. Bei der Überarbeitung lassen sich alte und neue FAQ-Versionen vergleichen (Überprüfungsmodus), sodass Änderungen nachverfolgt werden können. Im Dezember 2021 befinden sich in der Datenbank ca. 1000 FAQ, die ca. 450 Impf-FAQ (Abb. 2c) beinhalten und in regelmäßigen Abständen überarbeitet bzw. ergänzt werden. Tab. 2 fasst die Vor- und Nachteile einer einfachen Nutzung von Mutterdokumenten und einer Online-(Redaktions‑)Datenbank im FAQ-Aktualisierungsprozess zusammen.

**Table 2:** 

	Textverarbeitung mit (Mutter‑)Dokumenten und Tabellen	Onlinedatenbank/Tool (Sabio®)
*Vorhandene Vorkenntnisse*	Keine spezifischen Vorkenntnisse erforderlich	Softwareschulung der Beteiligten erforderlich
*Vorbereitungszeit*	Umsetzung schnell und simpel möglich	Intensive Vorbereitung und Implementierung erforderlich
*Anzahl FAQ*	Überblick geht schnell verloren, nur begrenzte Anzahl zu bearbeitender FAQ möglich	Anzahl der FAQ theoretisch unbegrenzt
*Anzahl beteiligter Institutionen*	Möglichst nicht mehr als 2 Parteien	Zusammenarbeit von mehr als 2 Parteien möglich
*Interner Austausch*	Einfacher und effektiver interner Austausch möglich	Für internen Austausch nicht erforderlich
*Austausch mit Auftragnehmern*	Guter Austausch mit externen Redaktionsteams und Agenturen möglich	Austausch nur nach Einweisung möglich

Eine wissenschaftliche Evaluation der Wirksamkeit der FAQ konnte bisher nicht umgesetzt werden, da aufgrund des dynamischen Geschehens der Pandemie die verfügbaren Kapazitäten v. a. für die Umsetzung der Maßnahmen genutzt wurden [20]. Es wurde jedoch konstant darauf geachtet, die von Brownson et al. [49] postulierten Kriterien für Evidenzbasierung zu berücksichtigen: die Nutzung aktueller, durch empirische Methoden generierter Erkenntnisse, die systematische Verwendung von Daten- und Informationssystemen sowie die Einbindung wichtiger Interessensvertreterinnen und -vertreter, Multiplikatorinnen und Multiplikatoren sowie Zielgruppen ([20, 49]; Infobox 2). Darüber hinaus wurden die Abrufzahlen der FAQ beobachtet, um das Interesse der Nutzerinnen und Nutzer an diesem Angebot einschätzen zu können.

### Analyse der FAQ-Nutzung auf Grundlage von Abrufzahlen

**Figure 3:**
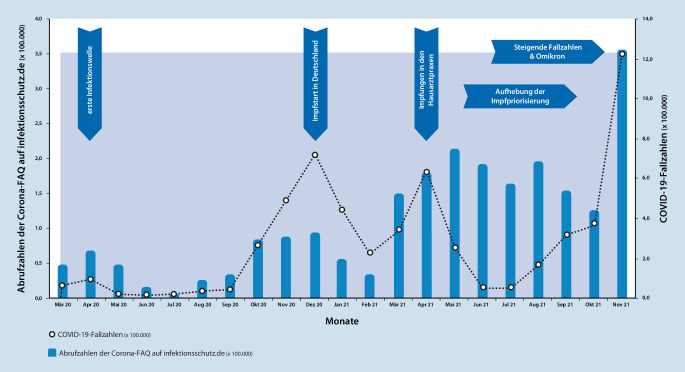

Die Abrufzahlen von infektionsschutz.de [25] werden durch Matomo Analytics, einer weitverbreiteten Open-Source-Webanalytik-Plattform [50] gezählt sowie dokumentiert und können daher ausgewertet werden. Abb. 3 gibt einen zeitlichen Überblick über die Abrufzahlen der FAQ-Seiten. Gleichzeitig sind die Fallzahlen positiver SARS-CoV-2-Tests dargestellt sowie Ereignisse, die eine mögliche Erklärung der veränderten Abrufzahlen sein können. Hier lassen sich grob parallele bzw. z. T. entsprechend leicht verzögerte Entwicklungen der Fallzahlen und der Abrufzahlen beobachten. Auch wenn hier keine detaillierten Auswertungen der Abrufzahlen mit den Infektionszahlen vorgenommen wurden, so scheinen die FAQ als Reaktionstool im Rahmen der Pandemie (Krisenreaktionsinstrument) insgesamt geeignet zu sein.

## Diskussion

Die FAQ der BZgA entwickeln sich in einem Zusammenspiel von Infektionsgeschehen und sich verändernden Informationsbedarfen der Bevölkerung und spezifischer Interessensgruppen sowie durch den Austausch und die Vernetzung mit den anderen Behörden. Dadurch wird gewährleistet, dass die jeweils aktuellsten verfügbaren wissenschaftlichen Erkenntnisse und Expertise sowie die Bedürfnisse der Bevölkerung im Sinne einer evidenzbasierten Vorgehensweise in die FAQ einfließen [49].

Die FAQ haben sich als ein effektives Format zur Informationsvermittlung in der Krise herausgestellt, um schnell und vielseitig auf das Infektionsgeschehen, die Anliegen der Bevölkerung und die politische Entwicklung zu reagieren. Sie zeichnen sich vor allem durch eine sachliche und transparente Vermittlung von Informationen und Handlungsempfehlungen aus, wodurch Vertrauen entstehen, die Bereitschaft, diese Informationen anzunehmen, steigen und die Wahrscheinlichkeit des entsprechenden Verhaltens erhöht werden kann [51]. Zudem wird in den FAQ mit persuasiven Botschaften gearbeitet, denn es wird betont, dass ein gewisses Verhalten eine positive oder negative Konsequenz für einen selbst oder andere mit sich bringen kann. Dies kann ebenfalls bewirken, dass die Bereitschaft, entsprechend zu handeln, steigt [52].

Die Aktualität der FAQ im dynamischen Infektionsgeschehen ist ein wichtiges Qualitätskriterium, auf das kontinuierlich geachtet wird, denn aktuelle und umfangreiche FAQ vermitteln nicht nur Wissen, sondern können auch das Vertrauen fördern [13]. Dazu gehört auch, dass die Inhalte innerhalb und zwischen den FAQ-Zweigen stimmig sein müssen. Dafür sind eine umfassende und stets aktuelle Dokumentation, ein ständiger und intensiver Austausch zwischen den Beteiligten aller Bereiche sowie regelmäßige Meetings erforderlich. Die Geschwindigkeit, mit der die Inhalte erstellt werden müssen, stellt dabei eine besondere organisatorische Herausforderung dar – insbesondere mit Blick auf die in der Pandemie begrenzten personellen Ressourcen [20]. Für die Zukunft ist es daher wichtig, den Austausch zwischen den Behörden weiter zu optimieren. Erkenntnisse und Entwicklungen müssen frühestmöglich kommuniziert bzw. angekündigt werden, sodass deren Bereitstellung für die Öffentlichkeit adäquat und effizient vorbereitet werden kann.

Um das Vertrauen zu fördern, ist es wichtig auch wissenschaftliche Unsicherheiten zu kommunizieren [20, 23]. Risikokommunikation, und das sind an dieser Stelle die FAQ, sollte die komplexen Prozesse der Wissenschaft und die dahinterstehenden Phänomene und Erklärungen für die Bevölkerung verständlich und nachvollziehbar erklären [53, 54]. Dies kann dann zur Akzeptanz etwaiger Empfehlungen und Maßnahmen beitragen und das Vertrauen in die kommunizierende Institution stärken.

Eine weitere Herausforderung in der Pandemie ist der Umgang mit Fehlinformationen (z. B. Impfmythen und Verschwörungstheorien), welche perspektivisch auch im Rahmen der FAQ mehr berücksichtigt werden sollten. Es erscheint erforderlich, Fehlinformationen stärker durch die Anwendung der Methodik des „Debunking“ zu entkräften [55]. Dabei werden Fehlinformationen zunächst identifiziert und anschließend entlarvt und richtiggestellt [56, 57].

Zudem stellt die Erreichbarkeit der Zielgruppen eine weitere Herausforderung dar. Einige Zielgruppen können mit FAQ nur schwer adressiert werden (z. B. Menschen mit Sprachbarrieren oder junge Menschen, die andere Plattformen zur Informationssuche präferieren bzw. eine zielgruppenspezifischere Kommunikation bevorzugen). FAQ eignen sich zudem insbesondere für Menschen, die sich selbst aktiv informieren [58–60]. Somit ist es wichtig, die FAQ in eine Kommunikationsstrategie zu integrieren, die sich auf mehrere Kanäle und unterschiedliche Instrumente erstreckt und sowohl Zielgruppen selbst als auch Multiplikatorinnen und Multiplikatoren anspricht, um eine möglichst hohe Reichweite zu erzielen [20].

Auch die Evaluation der FAQ stellt in der pandemischen Lage und aufgrund der dadurch bedingten begrenzteren Kapazitäten eine Herausforderung dar, sie konnte deswegen nicht optimal umgesetzt werden. Um dies zukünftig besser zu berücksichtigen, könnten z. B. auf der Internetseite simple Möglichkeiten für Feedback und Evaluation angeboten werden (z. B. kurze Onlineumfragen, Bewertungsmöglichkeit einzelner FAQ [hilfreich: Ja/Nein]). Um die Bedarfe der einzelnen Bevölkerungsgruppen noch besser zu verstehen und gleichzeitig die Bedarfserfüllung mittels der FAQ evaluieren zu können, sollte in Zukunft ein noch stärkerer Austausch mit den Zielgruppen und relevanten Multiplikatorinnen und Multiplikatoren stattfinden, beispielsweise mittels quantitativer Pre- und Posttests sowie qualitativer Fokusgruppen zu den FAQ. Zudem könnte die Auswertung von Social-Media-Daten (Social Listening) noch konsequenter berücksichtigt und genutzt werden [20].

## Fazit

Der Artikel beschreibt den Entwicklungs- und Veröffentlichungsprozess der FAQ zu COVID-19 der BZgA. Der Wandel der FAQ vom einfachen Informationsangebot zum interinstitutionellen Krisenreaktionsinstrument (Rapid Reaction Tool) wird dadurch deutlich. Hervorzuheben ist hierbei die enge Zusammenarbeit im Geschäftsbereich u. a. auf Grundlage von nationalen Plänen und Vorschriften [16–18] sowie Strategien [33], die eine kongruente, evidenzbasierte und tagesaktuelle Informationsbereitstellung auf einer einheitlichen Wissensdatenbasis für die Bevölkerung ermöglichen.

Die FAQ werden im Sinne einer Triangulierung aus verschiedenen Quellen gespeist (Bedarfe und Präferenzen der betroffenen Bevölkerungsgruppen, Expertise relevanter Expertinnen und Experten und evidenzbasierte Erkenntnisse) und basierend auf festgelegten Qualitätskriterien ([20]; Infobox 1a) formuliert, um valide und effektive Inhalte zu kreieren. Die FAQ sollen Vertrauen generieren, Wissen vermitteln, Skepsis und Ängste abbauen, Kompetenzen nachhaltig verankern und Selbstwirksamkeit stärken (vgl. Ziele der Risiko- und Krisenkommunikation). Die Förderung von Verhaltens- und Verhältnisprävention ist dabei ein wichtiges Ziel.

Die beschriebenen Prozesse dienen der Transparenz. Die dargestellten Schilderungen beinhalten Beispiele, wie FAQ angegangen und umgesetzt werden können. Gleichzeitig werden Lücken aufgedeckt und Empfehlungen für die Zukunft formuliert. So können die BZgA selbst und andere Institutionen, die FAQ bereitstellen, von den Erfahrungen der BZgA bzgl. der Nutzung der FAQ als Krisenreaktions- und Kommunikationsinstrument lernen und darauf aufbauen.

### Infobox 1 Anforderungen an FAQ-Inhalte (a) und Bedingungen für die Effektivität, Effizienz und Benutzerfreundlichkeit von FAQ (b) auf wissenschaftlicher Grundlage

Bei der Entwicklung der FAQ für die Bevölkerung wurden von der BZgA die folgenden Kriterien zugrunde gelegt:

- *FAQ-Inhalte müssen* [37–40]:
  fundiert, wissenschafts- bzw. evidenzbasiert sein,
  aktuell sein,
  für die Zielgruppen der BZgA relevant sein (i. d. R. die Allgemeinbevölkerung),
  klar verständlich formuliert sein, sodass auch fachferne Nutzerinnen und Nutzer diese gut verstehen können,
  dem Ziel der Wissensvermittlung entsprechen (informationsbasiert) und weder leitend noch meinungsorientiert sein,
  für alle Zielgruppen angemessen formuliert sein (z. B. Vertrauen aufbauen, Ängste und Sorgen abbauen).
- *Bedingungen für Effektivität, Effizienz und Benutzerfreundlichkeit von FAQ *[40]:
  prominente Platzierung der FAQ auf der Webseite; Unterseiten sollten auf die FAQ verlinken,
  Strukturierung der FAQ muss aus eindeutigen und selbsterklärenden, thematisch abgegrenzten Ober- und Unterkategorien bestehen,
  Beschränkung der Anzahl der FAQ pro Unterkategorie auf das Nötigste,
  Möglichkeit der Kontaktaufnahme für offene Fragen.

### Infobox 2 Inhaltliche Grundlagen der BZgA-FAQ, die unter Berücksichtigung der einzelnen Zielgruppen allgemeinverständlich aufgearbeitet werden

- *Evidenz aus der Forschung,* z. B. Umfrageergebnisse zu dem Wissens- und Gemütszustand der Allgemeinheit und spezifischer Bevölkerungsgruppen (z. B. COSMO-Daten [43], Ergebnisse der COVIMO- [44] sowie der CoSiD-Studie [45], BZgA-Studien zum Infektionsschutz [46], Empfehlungen der STIKO zur COVID-19-Impfung [47], Informationen aus dem RKI-Steckbrief [29], von der WHO und des European Centre for Disease Prevention and Control (ECDC))
- *Aktuelle politische Geschehnisse und Entscheidungen*, z. B. (Gesetzes‑)Beschlüsse und Verordnungen etc.
- *Aktuelle Nachrichtenlage* (Presse)
- *Bürgeranfragen* via E‑Mail, Telefon, Social Media, postalische Anfragen
- *Inhalte und Veröffentlichungen diverser Bundesbehörden,* z. B. des RKI, des BMG, des PEI und des BfArM, aber auch von der Bundesanstalt für Arbeitsschutz und Arbeitsmedizin (BAuA), dem Bundesamt für Bevölkerungsschutz und Katastrophenhilfe (BBK) sowie dem Friedrich-Loeffler-Institut (FLI) *und diverser Fachgesellschaften*
- *Besprechungen und Abstimmungen* auf Bundes- und Länderebene sowie BZgA-intern

## Footnotes

1 In der epidemiologischen Lagekonferenz (EpiLag) tauschen sich Vertreterinnen und Vertreter der zuständigen oberen Landesgesundheitsbehörden der 16 Bundesländer sowie der Bundeswehr und des RKI wöchentlich zur epidemiologischen Lage und zu weiteren Themen aus.

2 Bis zum 31.08.2021 hieß das entsprechende Fachreferat der BZgA „Referat 1-11 für medizinische Grundsatzfragen“. Durch eine Umstrukturierung der BZgA wurden die Themen des ursprünglichen Referats am 01.09.2021 in eigenständige Referate überführt. Das Thema Infektionsschutz wird vom „Referat T2 – Infektionsschutz, Risiko- und Krisenmanagement“ bearbeitet.

3 Die Kommunikationsagentur ist auf Grundlage eines nationalen Ausschreibungsverfahrens für die BZgA tätig und mit der technischen Betreuung und Weiterentwicklung der Online- und Printmedien zum Infektionsschutz betraut. Im Falle eines außergewöhnlichen infektiologischen Ausbruchsgeschehens kann sie von der BZgA in den Krisenmodus versetzt werden.

4 Die Unterstützung durch das externe Redaktionsteam trägt neben der Aktualität zur Qualität und Verständlichkeit der FAQ bei und gewährleistet die Einheitlichkeit von Sprache und Tonalität. Damit verbunden sind koordinierende Aufgaben zwischen den Mitgliedern des Redaktionsteams, die enge Abstimmung mit dem Fachreferat und der Kommunikationsagentur (für das Content-Management-System (CMS) und Erstellung von Printmedien zuständig).

5 Die Kommunikationsagentur begleitete das zuständige Fachreferat der BZgA bereits vor der Pandemie (wie auch das externe Redaktionsteam). Es wurde lediglich die im Vertrag vereinbarte und in Absatz 2 erläuterte Krisenoption gezogen.
